# Impact of influenza-like illnesses on health state utility value among Japanese children and adults

**DOI:** 10.1186/s41687-025-00917-x

**Published:** 2025-07-07

**Authors:** Taito Kitano, Shinya Tsuzuki

**Affiliations:** 1 AMR Clinical Reference Center, National Center for Global Health and Medicine, Japan Institute for Health Security, Tokyo, Japan; 2 Disease Control and Prevention Center, National Center for Global Health and Medicine, Japan Institute for Health Security, 1-21-1 Toyama, Shinjuku-ku, Tokyo, 162-8655 Japan; 3 Faculty of Medicine and Health Sciences, University of Antwerp, Antwerp, Belgium

**Keywords:** Influenza-like illness, Health-related quality of life, Population norm, Japan

## Abstract

**Purpose:**

For future health technology assessment, an assessment of the utility value of influenza-like illnesses (ILIs) is crucial. Therefore, the objective of this study was to evaluate the impact of ILIs on utility value in a Japanese population.

**Methods:**

We conducted an online survey between March and June 2024 to evaluate the impacts of ILIs on health-related quality of life, using a Japanese version of the EuroQol 5 Dimensions 5 Levels (EQ-5D-5 L) and EuroQol visual analog scale (EQ-VAS). Participants were children and adults aged < 80 years who experienced ILI symptoms or required home isolation due to a respiratory infection. A follow-up survey was conducted 2–3 weeks after the first survey to assess recovery. For children, we asked their parents or guardians to answer as the child’s proxy. A generalized linear model was used to assess the impact of patient demographics, type and onset of symptoms and diagnosis on disutility.

**Results:**

In total, 392 participants answered the first survey, and 264 participants answered the follow-up survey (134 adult participants and 130 parents or guardians). Compared with those who only answered the first survey, those who also answered the follow-up survey were older and more likely to be male in adult participants. The mean differences in the utility value and VAS scores between the first and follow-up surveys were − 0.055 and − 10.6 in the adult samples and − 0.079 and − 17.9 in the pediatric samples, respectively. In the generalized linear model, symptom onset within 7 days in the first survey was significantly associated with disutility value (coefficient − 0.049 [95% confidence interval [CI] − 0.086 to − 0.012], *p* = 0.010). However, none of the patient demographics were significantly associated with disutility value.

**Conclusion:**

Utility values were lower during the symptomatic phase compared with the recovery phase. Our results are useful for disease burden assessment, health technology assessment, and cost-effectiveness analysis, which can support decision-making on the preventive and therapeutic management of respiratory infections.

**Supplementary Information:**

The online version contains supplementary material available at 10.1186/s41687-025-00917-x.

## Abstract (plain language summary)

Influenza-like illnesses, the so-called common cold, are a non-negligible cause of disease burden worldwide. As quantitative disease burden assessments for these diseases have not been established in Japan, the aim of this study was to do it. Questionnaire surveys showed that health state utility value, a value representing a health state on a scale from 0 (death) to 1 (perfect health), was on average 0.055 and 0.079 lower during symptoms of influenza-like illnesses than after recovery in adults and children, respectively. The results of this study can be applied to the evaluation of future infectious disease control measures, such as vaccinaion policies, school closure, movement restrictions, and so forth.

**Supplementary Information:**

The online version contains supplementary material available at 10.1186/s41687-025-00917-x.

## Introduction

Respiratory infection is a major contributor to morbidity and mortality worldwide [1]. Due to its large impact on global disease burdens, the epidemiological surveillance of respiratory infections is crucial. Influenza-like illnesses (ILIs) are frequently used for the epidemiological surveillance of respiratory infection based on symptoms [2]. Thus, evaluation of the disease burdens of ILIs helps to elucidate the societal impacts of respiratory infections.

Health technology assessment (HTA) has increasingly been applied to inform health decision-making (e.g., introduction of new interventions, including preventive and therapeutic managements) [2]. This process includes cost-effectiveness and cost-utility analyses, which typically use quality-adjusted life years (QALYs) in their evaluations [3–5]. QALYs are calculated using a health-related quality of life (HRQL) questionnaire from which health state utility value [HSUV] associated with a health condition is obtained and then multiplied by the time (years) spent in the health condition, with the utility value typically presented as a single summary score between 0 and 1 [6]. Evaluations of the HRQL score or HSUV are fundamental for an appropriate implementation of HTA. However, such data are relatively scarce for ILIs. The COVID-19 pandemic stressed the importance of obtaining HSUV data on ILIs to guide preventive and therapeutic options for respiratory infection epidemics or pandemics.

Although numerous instruments have been used to assess HRQL, some centers recommend the EuroQol 5 dimensions (EQ-5D) instrument [7, 8]. The characteristics of the EQ-5D instrument include its ability to obtain “today’s” health status, instead of that of a “week” or “month”. Because health status dynamically changes on a daily basis among people experiencing ILI symptoms, EQ-5D is an appropriate option for measuring the impact of ILIs on HRQL.

The Japanese government has recently placed a high priority on HTA, including cost-effectiveness analyses using QALYs [9]. For the future HTA of respiratory infection in Japan, an assessment of the HSUV of ILIs in the Japanese population is crucial. Therefore, the objective of the present study was to evaluate the impact of ILIs on HRQL in a Japanese population using the EQ-5D instrument.

## Methods

### Study setting and sample collection

We conducted an online survey between March and June 2024 to evaluate the impacts of ILIs on HRQL, using the Japanese versions of the EuroQol 5 Dimensions 5 Levels (EQ-5D-5 L) and the EuroQol visual analog scale (EQ-VAS). The EQ-5D-5 L instrument measures generic health status in five levels using five dimensions: mobility, self-care, usual activities, pain/discomfort, and anxiety/depression. The survey participants were Japanese speakers who experienced ILI symptoms or required home isolation due to a respiratory infection. ILI symptoms were defined as the presence of fever, cough, or sore throat.

Participants were recruited from registered users of a Japanese marketing research company. The study comprised two parts: adult and pediatric parts. In each part, the first survey included those who had experienced ILI symptoms and/or isolated at home due to microbiological diagnosis of an ILI (e.g., COVID-19) on the day before the first survey. For those who answered the first survey, a follow-up survey was conducted 2 weeks after the first survey to assess recovery.

In the adult part, we conducted an online survey in March 2024 for Japanese adults aged 20–79 years. In the pediatric part, we conducted an online survey in May 2024 of Japanese children aged < 20 years who recently experienced an ILI by asking their parents or guardians to answer the EQ-5D-5 L questions as the child’s proxy. Generally, the child-specific EQ-5D-Y-3 L is used for children aged < 13 years due to the lack of validation of the pediatric 5-level version (i.e., EQ-5D-Y-5 L). However, we used the EQ-5D-5 L for all age groups in this study. Parents or guardians with more than one child were asked to answer for their youngest child.

The survey included questions on participants’ age, gender, residential region, household members, household income, educational background, type of symptoms (fever, cough, and sore throat), date of symptom onset, and diagnosis (COVID-19, influenza, and respiratory syncytial virus) made by a healthcare facility, if present. Fever was defined as 37.8 °C or higher in this study. Across all age groups, participant’s health status in the EQ-5D was converted into a single summary utility value score based on the Japanese value sets of the EQ-5D-5 L using a composite time trade-off [10]. Originally, utility value is presented as a single score between 0 and 1, with 0 being death and 1 being perfect health. Based on the Japanese value sets, answers of “11111” (the worst health status) and “55555” (the best health status) in the five dimensions of the EQ-5D instrument were converted to − 0.025 and 0.949, respectively [10]. The outcome of interest was disutility value, defined as the difference in utility value between the first survey and the follow-up survey. A negative disutility value indicated that the score was lower in the first survey than in the follow-up survey.

### Statistical analysis

Data from adult samples and pediatric samples were analyzed separately. Answers in each domain of the EQ-5D instrument, utility value and EQ-VAS score between symptomatic phase and recovery phase were compared using Wilcoxon’s signed rank tests. In addition, a generalized linear model was used to assess the impact of explanatory variables on disutility. Identity link function was selected given the nature of the disutility value. Explanatory variables included participant’s age (child vs. adult), gender (female vs. male), the education level of participants or guardians in pediatric cases (college graduate or higher vs. high school graduate or lower), the employment status of participants or guardians in pediatric cases (full-time vs. non-full-time), annual household income (< JPY 2 million, JPY 2–9.99 million, and ≥ JPY 10 million), diagnosis (COVID-19, influenza, or respiratory syncytial virus), and days after symptom onset (less than 7 days vs. 7 days or more). A *p*-value of < 0.05 was set as statistically significant. All statistical analyses were performed using R (ver. 4.4.0).

## Results

In total, 392 participants answered the first survey (207 adult participants and 185 parents or guardians). Of these, 264 participants answered the follow-up survey (134 adult participants and 130 parents or guardians). Participants’ demographic characteristics are presented in Table 1. The median age and female proportion of participants who answered both surveys and those who only answered to the first survey were 42 and 30 years (*p* < 0.001), and 29.1% and 45.2% (*p* = 0.030), respectively, for adult participants and 8 and 8 years (*p* = 0.783), and 46.9% and 54.5% (*p* = 0.431) for children, respectively. The median durations between the first and follow-up survey responses were 13.8 and 14.0 days for adult and pediatric participants, respectively. A higher proportion of adult participants who responded to both the first and follow-up survey responses experienced sore throat compared with those with the first survey response only (67.2% and 52.1% in those with both and only the first survey responses; *p* = 0.047). In addition, a higher proportion of pediatric participants with the first and follow-up survey responses experienced cough compared with those with the first survey response only (76.9% and 60.0% in those with both and only the first survey responses; *p* = 0.031). Conversely, the proportion with a diagnosis was significantly lower for influenza (13.4% and 28.8%; *p* = 0.012) and for respiratory syncytial virus (5.2% and 16.4%; *p* = 0.016) in adult participants and for COVID-19 (23.8% and 43.6%; *p* = 0.016) in pediatric participants. Duration between symptom onset and first survey, job status, education level, and household income were not significantly difference between those who responded to both survey and those who responded to the first survey only.

**Table 1:** Backgrounds of study participants

	Participants in adult part (N = 207)			Participants in pediatric part (N = 185)		
	Answering both surveys (N = 134)	Answering first survey only (N = 73)	*p*-value	Answering both surveys (N = 130)	Answering first survey only (N = 55)	*p*-value
Age, median [IQR]	42.0 [29.3–52.8]	30.0 [25.0–44.0]	< 0.001	46.0 [40.0–50.0]	45.0 [38.0–49.0]	0.237
Gender (female)	39 (29.1%)	33 (45.2%)	0.03	40 (46.9%)	18 (54.5%)	0.863
Child’s age, median [IQR]				8.0 [3.0–14.0]	8.0 [3.5–13.5]	0.783
Preschool age				42 (32.3%)	19 (34.5%)	0.954
Primary school age				36 (27.7%)	15 (27.3%)	
Junior high school age or older				52 (40.0%)	21 (38.2%)	
Child's gender (female)				61 (46.9%)	30 (54.5%)	0.431
Symptoms						
Fever	95 (70.9%)	43 (58.9%)	0.111	103 (79.2%)	38 (69.1%)	0.197
Cough	75 (56.0%)	31 (42.5%)	0.087	100 (76.9%)	33 (60.0%)	0.031
Sore throat	90 (67.2%)	38 (52.1%)	0.047	70 (53.8%)	36 (65.5%)	0.195
Diagnosis						
COVID-19	61 (45.5%)	30 (41.1%)	0.641	103 (79.2%)	24 (43.6%)	0.012
Influenza	18 (13.4%)	21 (28.8%)	0.012	100 (76.9%)	20 (36.4%)	0.183
Respiratory syncytial virus	7 (5.2%)	12 (16.4%)	0.016	70 (53.8%)	11 (20.0%)	0.58
Duration between symptom onset and first survey						
< 7 days	61 (45.5%)	31 (42.5%)	0.596	59 (45.4%)	24 (43.6%)	0.78
7–13 days	25 (18.7%)	18 (24.7%)		48 (36.9%)	23 (41.8%)	
≥ 14 days	48 (35.8%)	24 (32.9%)		23 (17.7%)	8 (14.5%)	
Duration between first and followup survey responses (days)	13.8 [13.5–14.0]			14.0 [13.9–14.1]		
Job status						
Full-time job	98 (73.1%)	51 (69.9%)	0.63	100 (76.9%)	45 (81.8%)	0.559
Education level						
College or university graduate	83 (63.8%)	49 (67.1%)	0.545	94 (72.3%)	43 (78.2%)	0.466
Annual household income (JPY)						
< 2 million	14 (10.4%)	12 (16.4%)	0.443	3 (2.3%)	2 (3.6%)	0.871
2–9.99 million	89 (66.4%)	44 (60.3%)		85 (65.4%)	36 (65.5%)	
≥ 10 million	31 (23.1%)	17 (23.3%)		42 (32.3%)	17 (30.9%)	

Abbreviations: IQR, interquartile range; JPY, Japanese Yen

Values are presented as number (percent) unless specified otherwise. Wilcoxon’s rank sum tests were conducted for continuous andordinary variables, and chi-square or Fisher’s exact tests were implemented for dichotomous variables

^a^ Parental or guardian demographics

The results of the EQ-5D are presented in Table 2. Among the 134 adult and 130 pediatric participants who answered both the first and follow-up surveys, their answers at symptomatic phase had significantly more problems compared with those at recovery phase across the all five domains, except for anxiety/depression adult participants (*p* = 0.099). The mean differences in the utility value and VAS score between the first and follow-up surveys were − 0.055 and − 10.6 in adult samples and − 0.079 and − 17.9 in pediatric samples, respectively. Of the 134 adult and 130 pediatric participants, 109 and 115 reported an ILI symptom in the first survey. In these participants, the mean differences in the utility value and VAS score between the first and follow-up surveys were − 0.052 and − 14.0 for adult participants and − 0.084 and − 18.5 for pediatric participants, respectively (Supplemental Table 1). In the 134 adult and 130 pediatric participants, 96 and 109 reported a complete resolution of symptoms in the follow-up survey. Among these participants, the mean differences in the utility value and VAS score between the first and follow-up surveys were − 0.052 and − 9.4 for adult participants and − 0.063 and − 16.8, respectively (Supplemental Table 2).

**Table 2:** Summary of EQ-5D and EQ-VAS scores among participants who answered both the first and follow-up surveys

	Adult part participants (N = 134)			Pediatric part participants (N = 130)		
	First survey	Follow-up survey	*p*-value	First survey	Follow-up survey	*p*-value
Mobility						
No problems	86 (64.2%)	111 (82.8%)		101 (75.4%)	124 (92.5%)	
Slight problems	37 (27.6%)	16 (11.9%)		18 (13.4%)	4 (3.0%)	
Moderate problems	7 (5.2%)	4 (3.0%)	< 0.001	5 (3.7%)	0 (0.0%)	< 0.001
Severe problems	3 (2.2%)	2 (1.5%)		1 (0.7%)	0 (0.0%)	
Unable	1 (0.7%)	1 (0.7%)		5 (3.7%)	2 (1.5%)	
Self-care						
No problems	102 (76.1%)	118 (88.1%)		95 (70.9%)	117 (87.3%)	
Slight problems	24 (17.9%)	11 (8.2%)		16 (11.9%)	5 (3.7%)	
Moderate problems	7 (5.2%)	3 (2.2%)	0.005	4 (3.0%)	2 (1.5%)	< 0.001
Severe problems	1 (0.7%)	2 (1.5%)		1 (0.7%)	0 (0.0%)	
Unable	0 (0.0%)	0 (0.0%)		14 (10.4%)	6 (4.5%)	
Usual activities						
No problems	76 (56.7%)	105 (78.4%)		90 (67.2%)	115 (85.8%)	
Slight problems	38 (28.4%)	21 (15.7%)		23 (17.2%)	12 (9.0%)	
Moderate problems	15 (11.2%)	5 (3.7%)	< 0.001	8 (6.0%)	1 (0.7%)	< 0.001
Severe problems	5 (3.7%)	2 (1.5%)		3 (2.2%)	0 (0.0%)	
Unable	0 (0.0%)	1 (0.7%)		6 (4.5%)	2 (1.5%)	
Pain/discomfort						
No problems	56 (41.8%)	85 (63.4%)	< 0.001	69 (51.5%)	109 (81.3%)	< 0.001
Slight problems	55 (41.0%)	38 (28.4%)		43 (32.1%)	20 (14.9%)	
Moderate	18 (13.4%)	9 (6.7%)		16 (11.9%)	1 (0.7%)	
Severe problems	4 (3.0%)	2 (1.5%)		1 (0.7%)	0 (0.0%)	
Unable	1 (0.7%)	0 (0.0%)		1 (0.7%)	0 (0.0%)	
Anxiety/depression						
No problems	83 (61.9%)	96 (71.6%)		101 (75.4%)	113 (84.3%)	
Slight problems	30 (22.4%)	22 (16.4%)		22 (16.4%)	15 (11.2%)	
Moderate	16 (11.9%)	8 (6.0%)	0.099	4 (3.0%)	1 (0.7%)	0.011
Severe problems	2 (1.5%)	5 (3.7%)		1 (0.7%)	1 (0.7%)	
Unable	3 (2.2%)	3 (2.2%)		2 (1.5%)	0 (0.0%)	
Utility value			< 0.001			< 0.001
Mean (SD)	0.798 (0.156)	0.853 (0.143)		0.818 (0.173)	0.897 (0.094)	
Median [IQR]	0.844 [0.676–0.939]	0.939 [0.823–0.939]		0.895 [0.752–0.939]	0.939 [0.895–0.939]	
Difference in utility values						
Mean (SD)	0.055 (0.134)			0.079 (0.157)		
Median [IQR]	0.041 [-0.138–0.000]			0.000 [-0.105–0.000]		
EQ-VAS score			< 0.001			< 0.001
Mean (SD)	61.1 (24.4)	71.8 (22.9)		67.4 (24.9)	85.3 (14.6)	
Median [IQR]	66.0 [41.3–80.0]	80.0 [61.0–89.8]		74.0 [50.0–89.3]	89.0 [79.3–95.8]	
Difference in EQ-VAS scores						
Mean (SD)	-10.6 (24.5)			-17.9 (23.7)		
Median [IQR]	6.0 [-23.8–2.5]			10.5 [-30.0–0.0]		

Abbreviations: EQ-5D, EuroQol 5 Dimensions; IQR, interquartile range; SD, standard deviation; VAS, visual analog scale

*p*-values were calculated by Wilcoxon’’s signed rank tests

In the generalized linear model (Table 3), symptom onset within 7 days in the first survey was significantly associated with disutility value (coefficient − 0.049 [95% confidence interval (CI)] − 0.086 to − 0.012], *p* = 0.010). However, all other explanatory variables investigated in this analysis were not significantly associated with disutility value, including participant’s age, gender, diagnosis, job status, education level, and annual household income.

**Table 3:** Generalized linear regression with identity link function to evaluate associations of explanatory variables with the disutility value

	Coefficient [95% CI]	*p*-value
Age		
Preschool age	−0.039 [− 0.118 to 0.039]	0.328
School age (< 20 years)	0.025 [− 0.044 to 0.095]	0.476
20–39 years	0.030 [− 0.041 to 0.101]	0.407
40–59 years	0.015 [− 0.058 to 0.089]	0.678
60–79 years	Reference	
Gender (female)	−0.006 [− 0.043 to 0.032]	0.77
Diagnosis		
COVID-19	0.031 [− 0.008 to 0.069]	0.116
Influenza	0.009 [− 0.037 to 0.055]	0.712
Respiratory syncytial virus	−0.016 [− 0.077 to 0.044]	0.593
Days between symptom onset and		
first survey		
Within 7 days (vs. ≥7 days)	−0.049 [− 0.086 to − 0.012]	0.01
Job status		
Full-time work (vs. non-full-time work)	−0.016 [− 0.061 to 0.029]	0.475
Education level		
College graduate or higher (vs. high school graduate or lower)	−0.009 [− 0.049 to 0.030]	0.643
Annual household income (JPY)		
< 2 million	−0.018 [− 0.100 to 0.063]	0.661
2–9.99 million	−0.024 [− 0.065 to 0.016]	0.236
≥ 10 million	Reference	

Abbreviations: EQ-5D, EuroQol 5 Dimensions; IQR, interquartile range; SD, standard deviation; VAS, visual analog scale

## Discussion

This study evaluated the impact of ILIs on HRQL using the EQ-5D and EQ-VAS in Japanese adults and children. Both children and adults showed reductions in utility values (mean disutility value − 0.079 and − 0.055 in children/adolescents and adults, respectively) in their symptomatic phase compared with their recovery phase. The magnitude of the mean disutility values observed in the total (0.067), adult (0.055), and pediatric (0.079) participants in this study were all larger than the minimally clinical important difference in Japan (0.044) [11]. A direct comparison of disutility values between adult and pediatric participants is not straightforward because of the differences in the measurement of utility values in adults vs. children and in self-reported vs. proxy reporting.

In our study, the demographics of adult participant who did not answer the follow-up survey was different in age and sex from those who answered both surveys. In addition, there were some differences in symptom and diagnosis between the completed and dropped populations. In Japan, patients with COVID-19 and influenza require home isolation even if they are asymptomatic, which could be one of reasons for a lower proportion of symptom presence and a higher proportion of certain infectious disease diagnosis in the dropped participants.

The magnitudes of the reductions in the utility value and VAS in participants reporting complete resolutions were similar to those in all study participants who answered both the first and follow-up surveys. Some individuals with an ILI and COVID-19 have prolonged symptoms, including a post-infectious cough and post-acute COVID-19 syndrome [12, 13]. Some studies reported that severe symptoms were associated with prolonged symptoms in a post-acute stage [14]. Our results may have been due to the fact that participants who had severe symptoms (a low HRQL score) at the first survey were less likely to show a resolution in the follow-up survey, whereas those who had a complete resolution may have had milder symptoms.

Data regarding the impacts of ILIs on HRQL using the EQ-5D instrument vary widely among previous studies. For example, a study in the UK reported that the mean utility value in individuals with an ILI was 0.48 on their worst day, using the EQ-5D, compared with 0.83 in their background period [15]. Similarly, another study using the EQ-5D instrument also showed that the mean utility value in individuals with an ILI was 0.34 on their worst day compared with 0.97 in their background period [16]. The large magnitude of their utility value reduction may have been due to the fact that these studies obtained utility values on the worst day of symptoms.

Notably, the QALY loss in an individual with an ILI is affected by not only the magnitude of the disutility value but also the duration of symptoms. In addition, the QALYs in a population are also affected by the incidence of ILIs. For example, with a mean disutility value of 0.067 for ILIs, a mean ILI duration of 10 days, and an ILI incidence of 1/person-year in a population, the annual QALY loss in the population would be 183.4/100,000 persons. The utility value data obtained in the present study would help to estimate the QALY loss of ILIs and respiratory infections and thereby support national decision-making processes on preventive and therapeutic management strategies through HTA.

In our study, < 7 days since symptom onset was significantly associated with a larger reduction in the utility value. A study in the US found utility values of 0.817, 0.877, and 0.896 at 3 days, 2 weeks, and 4 weeks since symptom onset, respectively, among unvaccinated COVID-19 cases compared with 0.930 at their baseline [17]. These findings indicate that days since symptom onset plays a critical role in the HRQL score in those experiencing ILI symptoms.

Our study showed that age, gender, and other participant demographics were not associated with the disutility value of ILIs. A study in the US revealed that the utility values based on the EQ-5D on day 3 after a SARS-CoV-2 positive test result were 0.916, 0.927, and 0.936 in individuals aged 18–34, 35–64, and ≥ 65 years, respectively [18]. A study in Spain found that disutility values associated with influenza were 0.45 (95% CI 0.40–0.49) in females and 0.41 (95% CI 0.35–0.46) in males, respectively [19]. Although age and gender are generally important factors affecting baseline utility values, disutility values for ILIs may not be greatly affected by these factors based on the results of our study, as well as on those of other studies [20].

There are some limitations to this study. The assessment of symptom severity could not be implemented in this study. There may have been a potential sampling bias because of targeting only registered users of a commercial online platform, self-selection bias, and bias associated with symptom severity or causal pathogen. There were some differences in demographics, presence of symptoms, and diagnosis between those with follow-up survey response and those with first survey response only. The results of follow-up survey answers of the drop-out participants may have been different from those of participants who completed the follow-up survey. We used the EQ-5D-5 L instrument for both adult and pediatric HSUV evaluations. Although evaluation by five levels may allow for a detailed assessment, the pediatric version of the EQ-5D-Y-5 L has not yet been validated in the Japanese population [21]. With the current version of the EQ-5D-Y-3 L, the results for the children in our study may have been slightly different [22]. There may also have been other coincidental events or conditions that affected the utility values in both the symptomatic and recovery phases.

In conclusion, our study evaluated the impact of ILIs on utility value. Utility values were lower during a symptomatic phase than during a recovery phase. The magnitude of the reduction in the utility value was not associated with participants’ age, gender, or other demographic characteristics. The results of our study will be useful for disease burden assessment, HTA, and cost-effectiveness analysis related to the preventive and therapeutic management of respiratory infections.

## Electronic supplementary material

Below is the link to the electronic supplementary material.

Supplementary Material 1

## Data Availability

All data used in the manuscript are derived from the results of a questionnaire survey conducted by a private company. The raw data are available from the corresponding author upon reasonable request. The authors have access to the raw data without the need for any special permission from the company because the raw data do not include any personal information that can be connected to the participants.
