# Supplementary material for: Impact of influenza-like illnesses on health state utility value among Japanese children and adults
Source: J Patient Rep Outcomes. 2025 Jul 7;9:83. doi: 10.1186/s41687-025-00917-x (PMC12234942; doi:10.1186/s41687-025-00917-x)
Supplement: Supplementary file 1 — Supplementary Material 1 [file 41687_2025_917_MOESM1_ESM.docx]

Supplementary Table 1. Sensitivity analysis of EQ-5D and EQ-VAS scores among participants who had symptoms of influenza-like illness at the first survey.

|  | Adult part participants (N=109) | | Pediatric part participants (N=115) | |
| --- | --- | --- | --- | --- |
|  | First survey | Follow-up survey | First survey | Follow-up survey |
| Utility value |  |  |  |  |
| Mean (SD) | 0.798 (0.150) | 0.851 (0.142) | 0.815 (0.389) | 0.899 (0.402) |
| Median [IQR] | 0.831 [0.670–0.939] | 0.939 [0.823–0.939] | 0.821 [0.000–0.939] | 0.939 [0.000–0.939] |
| Difference in utility values |  |  |  |  |
| Mean (SD) | -0.052 (0.132) | | -0.084 (0.143) | |
| Median [IQR] | -0.044 [-0.140–0.000] | | 0.000 [-0.057–0.000] | |
|  |  |  |  |  |
| EQ-VAS score |  |  |  |  |
| Mean (SD) | 62.6 (23.4) | 71.9 (21.9) | 67.0 (36.4) | 85.5 (39.4) |
| Median [IQR] | 67.0 [42.0–80.0] | 80.0 [60.0–89.0] | 55.0 [0.0–81.5] | 81.0 [0.0–92.5] |
| Difference in EQ-VAS scores |  |  |  |  |
| Mean (SD) | -9.3 (24.4) | | -18.5 (22.1) | |
| Median [IQR] | -6.0 [-23.0–3.0] | | -2.0 [-23.0–0.0] | |

Abbreviations: EQ-5D, EuroQol 5 Dimensions; IQR, interquartile range; SD, standard deviation; VAS, visual analog scale.

Supplementary Table 2. Sensitivity analysis of EQ-5D and EQ-VAS scores among participants who had complete resolution of symptoms at follow-up survey.

|  | Adult part participants (N=96) | | Pediatric part participants (N=109) | |
| --- | --- | --- | --- | --- |
|  | First survey | Follow-up survey | First survey | Follow-up survey |
| Utility value |  |  |  |  |
| Mean (SD) | 0.827 (0.127) | 0.879 (0.119) | 0.840 (0.421) | 0.903 (0.439) |
| Median [IQR] | 0.889 [0.737–0.939] | 0.939 [0.867–0.939] | 0.780 [0.000–0.939] | 0.895 [0.000–0.939] |
| Difference in utility values |  |  |  |  |
| Mean (SD) | -0.052 (0.134) | | -0.063 (0.116) | |
| Median [IQR] | -0.020 [-0.110–0.000] | | 0.000 [-0.045–0.000] | |
|  |  |  |  |  |
| EQ-VAS score |  |  |  |  |
| Mean (SD) | 65.8 (21.8) | 75.2 (21.3) | 70.8 (38.9) | 87.6 (43.0) |
| Median [IQR] | 70.0 [50.8–82.0] | 81.0 [64.8–90.0] | 50.0 [0.0–82.0] | 80.0 [0.0–92.3] |
| Difference in EQ-VAS scores |  |  |  |  |
| Mean (SD) | -9.4 (24.6) | | -16.8 (20.5) | |
| Median [IQR] | -6.0 [-22.3–1.5] | | 0.0 [-18.3–0.0] | |

Abbreviations: EQ-5D, EuroQol 5 Dimensions; IQR, interquartile range; SD, standard deviation; VAS, visual analog scale.
